# Stress distribution in three-unit implant-supported prostheses with different abutment designs: a finite element analysis

**DOI:** 10.1186/s12911-026-03690-z

**Published:** 2026-07-09

**Authors:** Ismail Furkan Guney, Busra Tosun

**Affiliations:** Department of Prosthodontics, Faculty of Dentistry, Bolu Abant Izzet Baysal University, Bolu, Turkey

**Keywords:** Dental implant, Finite element analysis, Multi-unit abutment, Stress distribution, Ti-base abutment

## Abstract

**Background:**

Implant-supported prostheses are widely used in posterior mandibular restorations, where biomechanical factors play a critical role in long-term performance. Abutment design may influence stress distribution within implant-supported systems, potentially affecting both prosthetic components and surrounding bone. This study aimed to evaluate stress distribution in three-unit implant-supported prostheses with different abutment designs using three-dimensional finite element analysis.

**Methods:**

A three-dimensional finite element model of the posterior mandible with two implants (4.1 mm diameter, 10 mm length) placed in the first premolar and first molar regions was constructed. Three abutment designs (multi-unit, octa, and variobase) supporting monolithic zirconia restorations were analyzed. Vertical (150 N) forces were applied perpendicular to the occlusal surfaces, and oblique (200 N at 45°) forces were applied in the buccolingual direction. Stress distribution in implants, abutments, screws, restorations, and surrounding bone was evaluated.

**Results:**

Stress distribution varied according to abutment design and loading condition. Variobase abutments showed higher stress concentrations in implants under both loading conditions, while octa abutments exhibited higher stress values in screws and restorations. Multi-unit abutments demonstrated relatively lower stress values in screws and restorations compared to other designs. Oblique loading produced higher stress levels than vertical loading across all components. Bone stress values increased under oblique loading conditions.

**Conclusions:**

Within the limitations of this finite element analysis, abutment design influenced stress distribution in implant-supported prostheses, and loading direction affected stress magnitude. These findings provide biomechanical insight into the behavior of different abutment designs in posterior implant-supported restorations.

## Background

Tooth loss may result in functional, aesthetic, and psychological consequences that negatively affect patients’ quality of life. Implant-supported prostheses are widely used for the rehabilitation of partially or completely edentulous patients due to their high success rates and long-term survival performance [1–3]. However, the accurate evaluation of biomechanical parameters is essential for the long-term success of these prostheses [4].

Mechanical loads generated during mastication are transferred from the prosthetic superstructure to the implant and surrounding tissues. The magnitude and direction of these forces may lead to uneven stress distribution, particularly in posterior regions where occlusal loads are higher [5]. Lateral forces, in particular, may induce high stress concentrations in the cervical region of implants, potentially contributing to microdamage at the bone–implant interface [6].

Abutments play a critical role in implant-supported prostheses by connecting the prosthetic structure to the implant and influencing load transfer within the system. Different abutment designs have been developed to improve biomechanical performance and reduce prosthetic complications [7]. In screw-retained implant-supported prostheses, the choice of abutment design is influenced by multiple factors, including interocclusal space, occlusion, and prosthetic configuration [8, 9]. These systems are widely preferred due to their biomechanical advantages and improved retrievability compared to cement-retained restorations [10–12]. Screw-retained implant-supported prostheses may be designed with different retention approaches, which can influence prosthetic configuration and load transmission [8].

Implant loss is often associated with bone resorption in the peri-implant region, which may be influenced by stress concentration patterns [13]. Therefore, understanding and predicting stress distribution around implants is critical for optimizing treatment outcomes. Advances in computational modeling and digital simulation techniques have enabled more precise evaluation of these biomechanical factors.

Finite element analysis (FEA) is a widely used computational method for evaluating stress distribution in implant systems. This method allows realistic modeling of complex structures and facilitates the analysis of force transmission under controlled conditions [14]. FEA offers several advantages, including precise geometric representation, control of variables, and non-invasive evaluation [15]. Three-dimensional FEA has been extensively applied in dentistry to simulate biomechanical behavior without the limitations of in vivo or in vitro studies [16, 17]. However, limitations such as cost and the requirement for technical expertise should be considered [18].

Monolithic zirconia has been widely used in implant-supported restorations due to its high fracture resistance, low plaque accumulation, and favorable esthetic properties [19]. Additionally, it has been shown that monolithic zirconia restorations maintain adequate fracture resistance even at reduced thicknesses, providing reliable load-bearing capacity [20]. The aim of this study was to evaluate stress distribution in three-unit implant-supported prostheses with different abutment designs (multi-unit, octa, and variobase) placed in the posterior mandible under vertical and oblique loading conditions using three-dimensional finite element analysis.

## Methods

In this study, two implants with a diameter of 4.1 mm and a length of 10 mm (implant system; Straumann, Basel, Switzerland) were placed in the mandibular posterior first premolar and first molar regions with an 8° angle between them. All abutments were standard prefabricated components without additional angulation. The 8° inclination refers solely to the placement angle of the implants relative to the mandibular plane, not to the abutment design.

Three different abutment designs (multi-unit, octa, and variobase) were evaluated. Accordingly, three models were created, and monolithic zirconia restorations were placed on these abutments. Vertical and oblique forces were applied, resulting in six different study groups (Table 1). Stress values in the implant, abutment, screw, restoration, and surrounding bone were evaluated using linear finite element analysis.

**Table 1:** Study design and loading conditions for each model

Models	Abutment	Loading conditions
1	Multi-unit Multi-unit	Vertical loading
2		Oblique loading
3	Octa Octa	Vertical loading
4		Oblique loading
5	Variobase Variobase	Vertical loading
6		Oblique loading

### Preparation of work models

The geometric model of the mandibular region was obtained from an existing dataset available in the software database commonly used for finite element analyses. CBCT scanning was performed at 120 kVp and 3.8 mA, producing 601 slices with a thickness of 0.2 mm. The DICOM data were processed through segmentation using imaging software (3D-Doctor; Able Software, Lexington, MA, USA) and converted into a three-dimensional model.

The implant, abutment, and screw geometries were generated based on manufacturer specifications and scanned using an optical scanner (Activity 880; Smart Optics, Bochum, Germany) to obtain three-dimensional images. These models were transferred in STL format to modeling software (Rhinoceros 4.0; Robert McNeel & Associates, Seattle, WA, USA), where alignment between prosthetic and bone structures was performed and preparation for analysis was completed.

The teeth numbered 44, 45, and 46 in the posterior mandible region were included in the study. Additional mesh refinement was performed to improve element quality, particularly in the implant–abutment interfaces and occlusal contact regions. Contact interactions between all components were defined as bonded to ensure uniform load transfer across interfaces.

**Fig. 1:**
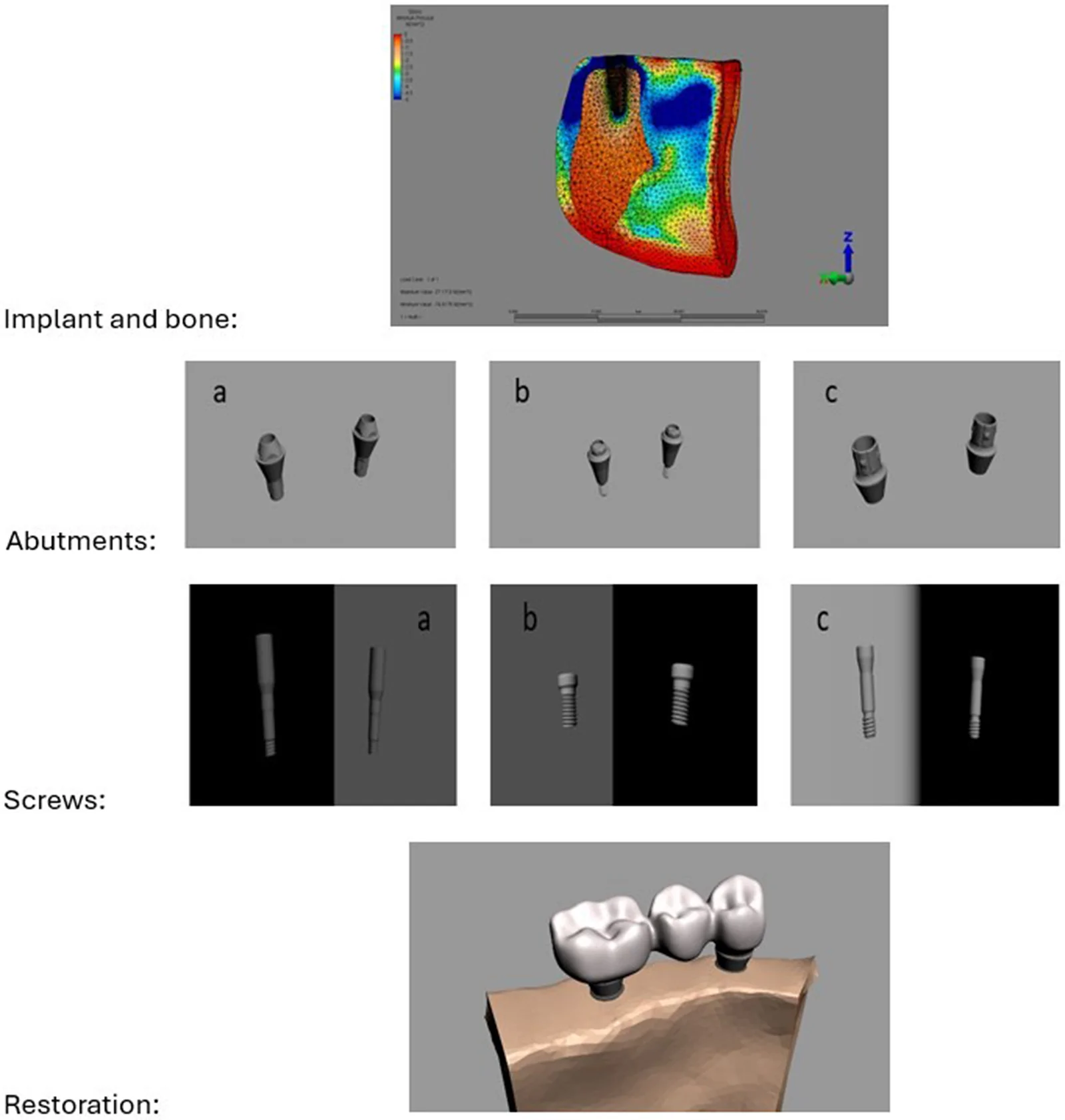
Components of the study: implant and bone segment, three abutment configurations with screws (**a**, multi-unit; **b**, octa; **c**, variobase), and the restoration

To ensure anatomical accuracy, tooth size and contour images obtained from the Wheeler atlas were scaled [21], and geometric modeling was performed using the same software environment. Monolithic zirconia (Bruxzir Solid Zirconia; 3 M ESPE, USA) was used for crown restorations (Fig. 1). Prosthetic restorations were digitally designed using dental CAD software (Exocad DentalCAD; exocad, Darmstadt, Germany) prior to mesh generation and analysis.

In the multi-unit configuration, the screw corresponds to the occlusal screw retaining the prosthesis, whereas in the variobase configuration, it represents the abutment screw fixing the abutment to the implant. All abutments and restorations in this study were screw-retained, and no cemented components or cement gaps were used.

## The creation of mathematical models

The geometric models were discretized into finite elements to create a numerical model. Mesh generation and optimization were performed using mesh processing software (VRMesh; VirtualGrid, Bellevue, WA, USA), and the models were prepared for analysis in finite element software (Algor Fempro; Autodesk, San Rafael, CA, USA).

After importing the STL files into Algor Fempro, all geometries were converted into volumetric solid meshes consisting of tetrahedral elements to allow accurate stress computation. Material properties of the mandible and other structures were assigned in the software environment. The Young’s modulus and Poisson’s ratio values listed in Table 2 were adopted from previously published data [22–24]. In accordance with a widely accepted finite element modeling guideline [25], the mesh density was set to exceed 30,000 nodes and 200,000 elements to ensure sufficient numerical accuracy and convergence. The number of nodes and elements used in this study are presented in Table 3.

**Table 2:** Mechanical properties of materials used in the finite element model

Material	Young’s modulus (MPa)	Poisson’s ratio (ν)
Titanium	110.000	0.35
Cancellous bone	1.370	0.30
Cortical bone	13.700	0.30
Monolithic zirconia	210.000	0.30
Resin cement	18.600	0.28

**Table 3:** Number of elements and nodes in the finite element models

Abutment type	Number of elements	Number of nodes
Multi-unit	738.280	146.630
Octa	446.216	92.099
Variobase	644.932	139.548

Mesh optimization was performed using adaptive mesh refinement to improve accuracy in regions with high stress gradients. Boundary conditions were defined by fully constraining the inferior and posterior surfaces of the mandibular bone model in all degrees of freedom, simulating fixation to surrounding anatomical structures. The implant–bone interface was modeled as fully bonded to represent complete osseointegration without micro-movement. All interfaces between components were assumed to be perfectly bonded, and no sliding or separation was permitted during loading.

Mesh refinement and convergence verification were conducted to achieve stable stress results and reduce numerical errors, in line with previous studies evaluating stress distribution in implant-supported crowns using finite element methods [26]. All materials were considered homogeneous, isotropic, and linearly elastic to allow consistent evaluation of stress distribution. All abutment screws were assumed to be tightened to 35 Ncm according to manufacturer recommendations, using calibrated torque-limiting devices to ensure accurate preload application [27].

## Loading conditions

**Fig. 2:**
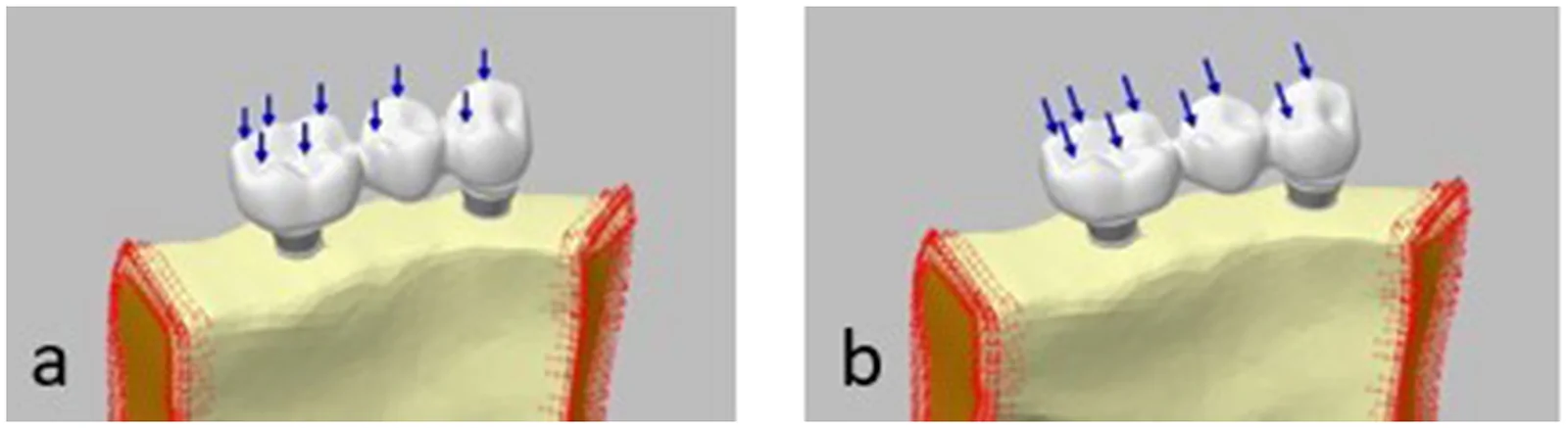
Direction of the applied forces: (**a**) vertical and (**b**) oblique loading

In this study, two different loading conditions were applied: vertical and oblique forces. According to the literature, the average occlusal force is approximately 150 N in the premolar region and 200 N in the molar region [28, 29]. Accordingly, 150 N was applied at two contact points on the first premolar and 200 N at five contact points on the first molar (Fig. 2).

Vertical forces were applied perpendicular to the occlusal surfaces, whereas oblique forces were applied at a 45° angle to the implant’s long axis in the buccolingual direction to simulate masticatory dynamics [30].

## Results

All stress values obtained in this study are presented in MPa for clarity. Differences in stress distribution were observed among the different abutment designs in the implant, its components, and surrounding bone (Table 4).

**Table 4:** Maximum von Mises stress values in implant components and bone under different abutment designs and loading conditions (MPa)

		Vertical loading		Oblique loading	
		Premolar	Molar	Premolar	Molar
Implant	Multi-unit	145.2	189.0	467.2	473.7
	Octa	126.8	215.8	249.0	464.5
	Variobase	239.3	255.8	604.6	667.5
Bone maximum principal stress	Multi-unit	1.1	4.0	15.4	21.1
	Octa	1.9	3.2	14.3	17.4
	Variobase	2.4	4.1	14.2	20.4
Bone minumum principal stress	Multi-unit	-30.7	-41.4	-60.8	-91.3
	Octa	-30.5	-42.9	-62.3	-91.2
	Variobase	-30.9	-41.7	-64.9	-89.1
Abutment	Multi-unit	152.0	199.8	509.9	511.6
	Octa	128.0	142.5	323.8	360.5
	Variobase	124.9	164.2	426.6	437.8
Screw	Multi-unit	136.8	143.7	91.8	157.2
	Octa	106.9	188.4	150.3	210.0
	Variobase	64.6	86.1	161.2	175.7
Restoration	Multi-unit	45.2	57.6	112.0	130.5
	Octa	97.3	115.5	242.1	365.0
	Variobase	52.5	73.4	155.1	191.0

Under vertical loading conditions, the highest von Mises stress values on the implant were observed in the variobase abutment models, reaching 239.3 MPa in the first premolar region and 255.8 MPa in the first molar region. Under oblique loading, stress values increased in all models, with the highest values again observed in the variobase abutment models (604.6 MPa in the first premolar and 667.5 MPa in the first molar regions).

**Fig. 3:**
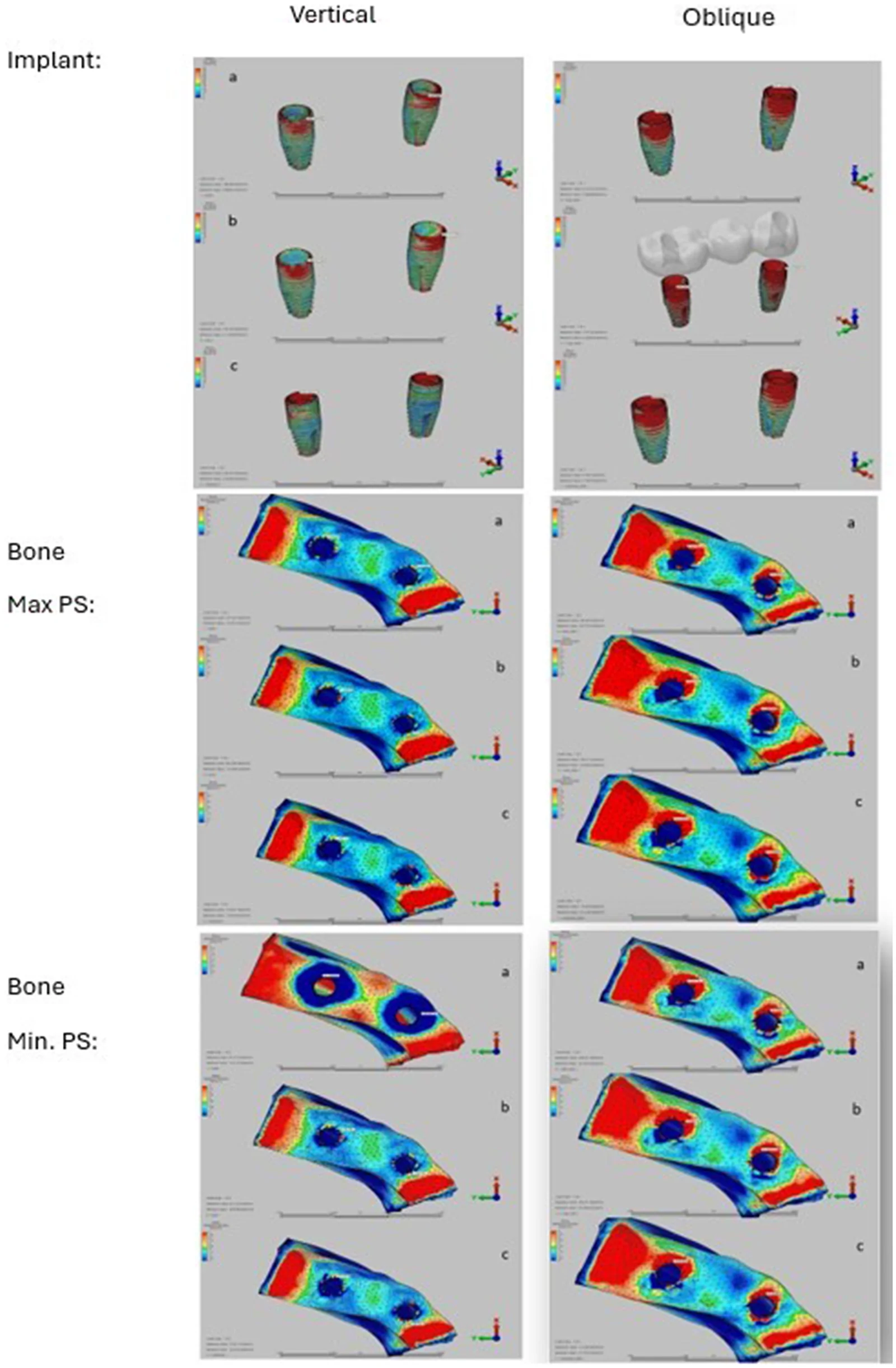
Distribution of von Mises stress in the implant and maximum and minimum principal stresses in the bone under vertical and oblique loading conditions for different abutment designs: (**a**) multi-unit, (**b**) octa, and (**c**) variobase

Stress distribution in bone varied according to abutment design and loading condition. Under vertical loading, the highest maximum principal stress values were observed in the variobase abutment models (2.4 MPa in the first premolar and 4.1 MPa in the first molar regions). Under oblique loading, the highest values were observed in the multi-unit abutment models (15.4 MPa in the first premolar and 21.1 MPa in the first molar regions). For minimum principal stress, the highest values under vertical loading were observed in the variobase abutment model in the first premolar region (30.9 MPa) and in the octa abutment model in the first molar region (42.9 MPa). Under oblique loading, the highest values were observed in the variobase abutment model in the first premolar region (64.9 MPa) and in the multi-unit abutment model in the first molar region (91.3 MPa) (Fig. 3).

Regarding abutment stress, the highest von Mises stress values under vertical loading were observed in the multi-unit abutment models (152.0 MPa in the first premolar and 199.8 MPa in the first molar regions). Under oblique loading, the highest stress values were observed in the variobase abutment models (509.9 MPa in the first premolar and 511.6 MPa in the first molar regions).

For screw stress, the lowest von Mises stress values under vertical loading were observed in the variobase abutment models (64.6 MPa in the first premolar and 86.1 MPa in the first molar regions). Under oblique loading, the lowest stress values were observed in the multi-unit abutment models (91.8 MPa in the first premolar and 157.2 MPa in the first molar regions).

**Fig. 4:**
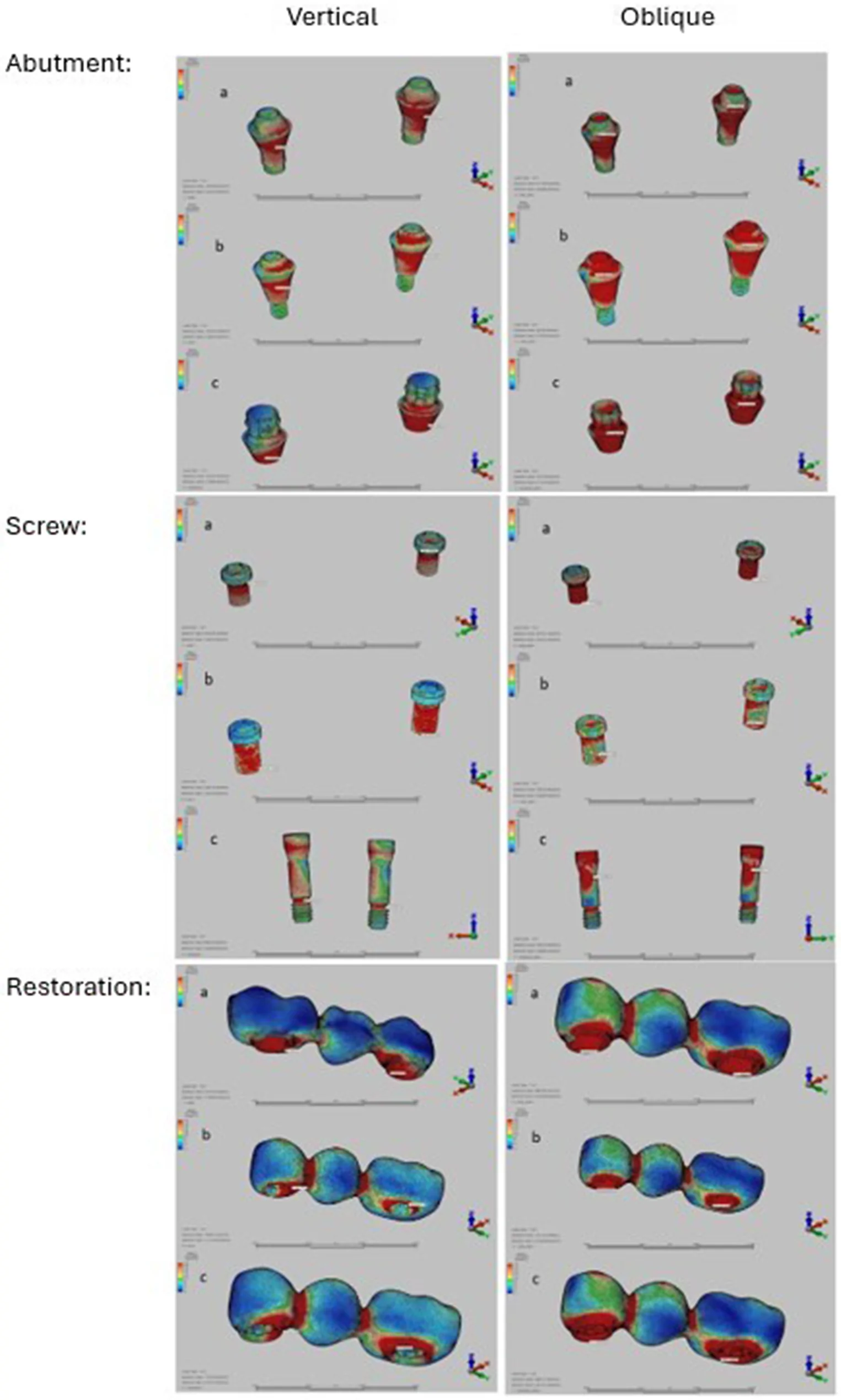
Distribution of von Mises stress in the abutment, screw, and restoration under vertical and oblique loading conditions for different abutment designs: (**a**) multi-unit, (**b**) octa, and (**c**) variobase

Restoration stress values also varied depending on abutment design and loading condition. Under vertical loading, the highest values were observed in the octa abutment models (97.3 MPa in the first premolar and 115.5 MPa in the first molar regions). Under oblique loading, the highest values were observed in the variobase abutment models (242.1 MPa in the first premolar and 365.0 MPa in the first molar regions) (Fig. 4).

## Discussion

Different abutment designs resulted in distinct stress distribution patterns across implant components and surrounding tissues. Variobase abutments produced higher stress concentrations in implants, approximately 50% greater than those observed in octa and multi-unit abutments. In contrast, multi-unit abutments showed higher stress values within the abutment structure, whereas octa abutments demonstrated increased stress accumulation in screws. These findings suggest that stress is redistributed differently depending on the abutment configuration rather than being uniformly transferred throughout the system. This observation is consistent with previous finite element studies reporting that non-engaging abutments may increase stress concentrations at the implant–abutment interface and prosthetic screws under oblique loading conditions [31].

Dental implants are widely used due to their long-term success in the rehabilitation of missing teeth [32]. Abutment design influences load transfer within implant-supported prosthetic systems and may affect biomechanical stability. Internal connection systems have been reported to reduce micro-movement and micro-leakage compared to external connections [33], and therefore internal connection abutments were selected. In clinical practice, ideal implant positioning is not always achievable. Tang et al. [34] reported an angular deviation between planned and placed implants, and a similar angulation (8°) was incorporated into the model to better simulate clinical conditions.

The inability to achieve passive fit in screw-retained implant-supported prostheses may lead to various mechanical complications [35]. It has been reported that hexagonal abutments may be insufficient in achieving passive fit, particularly when implants are not perfectly parallel [31], and that abutments without anti-rotational components may reduce technical complications [36]. Accordingly, non-hex, octa, and multi-unit abutment designs were preferred.

Because masticatory forces often act obliquely rather than parallel to the implant axis, finite element analyses commonly apply inclined loads to simulate clinical conditions [37]. Oblique forces were therefore applied at a 45° angle in accordance with the literature [38]. Occlusal contact points were determined based on Okeson’s reference points, with five points defined for molars and two for premolars [39]. Chewing forces vary among individuals and are influenced by factors such as age, gender, edentulous regions, existing restorations, and parafunctional habits [40]. Previous studies have reported average occlusal forces of approximately 200 N in the molar region and 150 N in the premolar region [28, 29], and these values were adopted in the model.

Monolithic zirconia was selected as the superstructure material due to its favorable mechanical properties and widespread use in implant-supported fixed prostheses [41]. Previous studies have demonstrated its mechanical reliability under functional loading conditions [19] and its high load-bearing capacity when used with multi-unit abutments [42]. Accordingly, restorations were designed to be directly screw-retained onto the abutments.

It has been reported that oblique forces generate higher stress concentrations in bone tissue, particularly at the implant neck [43]. Consistent with this, oblique loading produced higher stress values than vertical loading, especially in the implant neck and abutment regions. These findings confirm that non-axial loading plays a critical role in increasing biomechanical stress within implant-supported systems.

Although monolithic zirconia restorations demonstrate favorable long-term performance, restoration fractures remain a common complication [44]. Previous studies have shown that connector thickness significantly influences restoration strength, with thinner connectors increasing the risk of fracture [45]. Stress concentrations were primarily located in the cervical and connector regions of the restorations, supporting the importance of these areas in biomechanical failure. Among the evaluated models, octa abutments showed the highest stress values, whereas multi-unit abutments demonstrated lower stress levels. The higher stress observed in octa abutment models suggests a greater mechanical load concentration in the prosthetic structure.

Micron-level movements at the implant–abutment interface have been associated with increased stress concentrations in cervical implant regions [46]. The highest von Mises stress values were concentrated in the cervical region of the implant and at the implant–abutment interface, confirming that these areas represent critical zones for stress accumulation. Variobase abutments demonstrated a marked increase in stress values, whereas octa abutments showed comparatively lower stress levels at the implant level. These findings indicate that abutment design directly influences how stress is distributed between implant components.

High stress concentrations were also observed at the abutment–screw connection regions, consistent with previous studies [47, 48]. While octa abutments generally exhibited lower stress values, multi-unit abutments showed relatively higher stress accumulation in these regions, suggesting a redistribution of stress toward the connection interface. These findings highlight the importance of connection design in the biomechanical behavior of implant-supported prostheses.

Internal connection abutments (octa and variobase) were generally associated with lower stress values in peri-implant bone compared to alternative configurations. This observation is consistent with Chun et al. [49], who reported that internal connection systems reduce stress concentrations under inclined loading through improved load transfer at the implant–abutment interface.

Finite element analysis enables evaluation of stress distribution patterns across different abutment designs under controlled loading conditions. The observed variations in stress distribution indicate that abutment configuration directly influences biomechanical behavior within implant-supported systems.

In implant-supported restorations, screws are considered the weakest component and are frequently associated with complications such as loosening or fracture [50]. The magnitude of occlusal forces and the mechanical properties of implant components contribute to these complications [51]. Doğuş et al. [52] also reported that the load-bearing capacity of screws is limited. Higher stress values in abutment screws were observed in octa abutments, whereas lower stress values were identified in variobase abutments under vertical loading and in multi-unit abutments under oblique loading. These findings indicate that abutment design influences the distribution of stress within the screw component and may affect mechanical stability.

Occlusal loads are transmitted to the surrounding bone through the implant, and stress distribution is influenced by the mechanical properties of cortical bone [53]. Cortical bone exhibits greater resistance to stress due to its higher elastic modulus compared to cancellous bone [54]. Reported tensile and compressive limits of cortical bone are 121–135 MPa and 167–205 MPa, respectively [55]. The stress values observed remained within these physiological limits in all models, suggesting that the applied loading conditions did not exceed the biomechanical tolerance of bone tissue. However, higher stress values under oblique loading indicate that non-axial forces may contribute to increased biomechanical loading in peri-implant bone.

The maximum stress values observed in the restorations remained below the reported flexural strength of monolithic zirconia (800–1000 MPa) and the yield strength of titanium alloys commonly used for implants (> 800 MPa), indicating a favorable safety margin against mechanical failure [19, 56]. These findings suggest that the evaluated abutment configurations did not exceed the mechanical limits of the materials under the applied loading conditions.

Finite element analysis allows controlled evaluation of stress distribution under simulated conditions; however, it cannot fully replicate the complex biological environment of in vivo situations [57]. Therefore, the findings should be interpreted as indicative of biomechanical trends rather than exact clinical outcomes, and differences in tissue behavior may occur under clinical conditions [58]. The main limitations of this study include the simplified assumptions inherent to computational modeling and the inability to fully reproduce in vivo conditions. Accordingly, the results should be interpreted with caution and supported by further experimental and clinical investigations. Future research may focus on evaluating the effects of variations in implant diameter, angulation, and occlusal loading conditions, as well as different material properties, on stress distribution. Further investigation of these parameters may provide a more comprehensive understanding of biomechanical behavior in implant-supported prosthetic systems.

## Conclusion

Abutment design influenced stress distribution patterns across implant components and surrounding structures. Variobase abutments were associated with higher stress concentrations in implants, whereas octa abutments showed increased stress in screws and restorations. Multi-unit abutments demonstrated a more favorable distribution of stress within the prosthetic system. Oblique loading consistently resulted in higher stress values than vertical loading, indicating the importance of load direction in implant biomechanics.

## Data Availability

Data is provided within the manuscript.

## Abbreviations

CBCT: Cone beam computed tomography
DICOM: Digital Imaging and Communications in Medicine
FEA: Finite element analysis
MPa: Megapascal
N: Newton
Ncm: Newton centimeter
STL: Standard tessellation language
CAD: Computer-aided design
